# Convergent synthesis of ^13^N-labelled Peptidic structures using aqueous [^13^N]NH_3_

**DOI:** 10.1186/s41181-017-0035-7

**Published:** 2017-12-19

**Authors:** Julia E. Blower, Samuel F. Cousin, Antony D. Gee

**Affiliations:** King’s College London, School of Biomedical Engineering and Imaging Sciences, 4th Floor Lambeth Wing, St. Thomas’ Hospital, London, SE1 7EH UK

**Keywords:** PET chemistry, Nitrogen-13, [^13^N]ammonia, Multicomponent reaction, Peptide, Ugi

## Abstract

**Background:**

Nitrogen-13 has a 10-min half-life which places time constraints on the complexity of viable synthetic methods for its incorporation into PET imaging agents. In exploring ways to overcome this limitation, we have used the Ugi reaction to develop a rapid one-pot method for radiolabelling peptidic molecules using [^13^N]NH_3_ as a synthetic precursor.

**Methods:**

Carrier-added [^13^N]NH_3_ (50 μL) was added to a solution of carboxylic acid, aldehyde, and isocyanide in 2,2,2-TFE (200 μL). The mixture was heated in a microwave synthesiser at 120 °C for 10 min. Reactions were analysed by radio-HPLC and radio-LCMS. Isolation of the target ^13^N–labelled peptidic Ugi compound was achieved via semi-preparative radio-HPLC as demonstrated for Ugi**1.**

**Results:**

Radio-HPLC analysis of each reaction confirmed the formation of radioactive products co-eluting with their respective reference standards with radiochemical yields of the crude products ranging from 11% to 23%. Two cyclic γ-lactam structures were also achieved via intra-molecular reactions. Additional radioactive by-products observed in the radio-chromatogram were identified as ^13^N–labelled di-imines formed from the reaction of [^13^N]NH_3_ with two isocyanide molecules. The desired ^13^N–labelled Ugi product was isolated using semi-preparative HPLC.

**Conclusion:**

We have developed a one-pot method that opens up new routes to radiolabel complex, peptidic molecules with ^13^N using aqueous [^13^N]NH_3_ as a synthetic precursor.

**Electronic supplementary material:**

The online version of this article (10.1186/s41181-017-0035-7) contains supplementary material, which is available to authorized users.

## Background

Nitrogen-13 (t_1/2_ = 9.97 min; 100% β^+^ decay) is used clinically in the field of positron emission tomography (PET). Compared to other cyclotron-produced positron emitters such as ^18^F and ^11^C, ^13^N has been largely overlooked as a viable option for radiolabelling as its short half-life poses challenges to the development of synthetic methods. Despite its limited use, ^13^N offers many favourable attributes that may be complementary to conventional ^11^C and ^18^F labelling: the ubiquity of nitrogen in endogenous molecules and pharmaceuticals allows direct labelling of molecules of interest without interfering with their biological activity, enabling study of authentic biogenic molecules, and the short half-life offers the ability to repeat PET scans on the same individual within a short time and without excessive radiation dose to the patient. Cyclotron-produced ^13^N is most commonly generated by the proton bombardment of [^16^O]H_2_O via the nuclear reaction ^16^O(p,α)^13^N to produce [^13^N]NH_3_ (Wieland et al., 1991), which is the only routinely-used ^13^N radiopharmaceutical (for myocardial perfusion imaging) but can also be a potentially useful radiolabelling precursor (Kumata et al., 2010; Kumata et al., 2012; Gomez-Vallejo et al., 2014).

Multi-component reactions are powerful tools in the efficient and rapid generation of diverse libraries of compounds, and are particularly useful for high-throughput screening. The four-component Ugi reaction combines a carboxylic acid, an amine (sometimes ammonia), an aldehyde or ketone, and an isocyanide to create α-aminoacyl amide derivatives - peptidomimetic structures with proven pharmaceutical application (Domling & Ugi, 2000). Notably, the one-pot synthesis of the local anaesthetic Xylocain and the subsequent development and marketing of at least 12 other anaesthetics is based on this scaffold (Hulme & Gore, 2003). The Ugi reaction is therefore an attractive method for the rapid radiolabelling of peptides with short-lived PET radionuclides, useful in diagnosis, drug discovery and as a research tool in understanding molecular mechanisms in vivo. Here we report the radiolabelling of α-aminoacyl amide derivatives with ^13^N, via the four-component coupling (4-CC) Ugi synthesis, using aqueous [^13^N]NH_3_ as a precursor.

## Methods

### General

Benzaldehyde (≥99%), levulinic acid (98%), *p*-toluic acid (98%), tert-butyl isocyanide (98%) and trifluoroacetic acid (99%) were purchased from Sigma-Aldrich. Benzyl isocyanide (98%) and 2,2,2-trifluoroethanol (99.8%) were purchased from Acros Organics. 1,1,3,3-tetramethylbutyl isocyanide and ammonia solution (28–30%) were purchased from Merck KGaA. Acetic acid (~100%) was purchased from AnalaR. Methanol (HPLC grade) was purchased from Fisher.

Reactions were carried out using a CEM Discover microwave synthesis unit.

^1^H–NMR and ^13^C–NMR spectra were obtained using a Bruker DRX 400 MHz spectrometer. Mass spectrometry was performed on an Agilent Technologies 6520 Accurate-Mass Q-TOF LC/MS connected to an Agilent Technologies1200 HPLC system with UV detector and autosampler.

Radio-HPLC analysis was performed on an Agilent Technologies 1200 Series with UV detector (254 nm) and Lablogic β + radio-detector using an Agilent Eclipse XDB-C18 column (5 μm, 4.6 × 150 mm). The following mobile phase conditions were used: solvent A: H_2_O + 0.1% TFA; solvent B: MeOH +0.1% TFA; time:%B 0:5, 1:5, 10:95, 18:5, 23:5. Flow rate was 1 mL/min.

### Reference standard synthesis

For reference standard compounds **1–5,** benzaldehyde (2 mmol), the respective carboxylic acid (*p*-toluic acid (**2, 4**) or acetic acid (**1, 3, 5**), 2 mmol), the respective isocyanide (tert-butyl isocyanide (**4, 5**), benzyl isocyanide (**3**) or 1,1,3,3-tetramethylbutyl isocyanide (**1, 2**), 2 mmol) and ammonia (excess, ~ 3 mmol) were combined in 2,2,2-trifluoroethanol (TFE, 2 mL) in a microwave tube. For reference standard compounds **6–7**, levulinic acid (2 mmol), the respective isocyanide (benzyl isocyanide (**6**) or tert-butyl isocyanide (**7**), 2 mmol) and ammonia (excess, ~ 3 mmol) were combined in TFE (2 mL) in a microwave tube. The mixture was heated at 100 °C for 30 min in a microwave synthesis unit. The reaction mixture was cooled to room temperature and the solvent was removed under vacuum. The crude product was filtered and washed with ice-cold methanol. The final product was isolated using semi-preparative HPLC and lyophilised.

### Radiochemistry

#### [^13^N]NH_3_ production

Aqueous [^13^N]NH_3_ was produced on a CTI RDS 112 biomedical cyclotron via the ^16^O(p,α)^13^N nuclear reaction. The target contained 8 mL H_2_O with 5 mM ethanol. Concentration of [^13^N]NH_3_ into a 1 mL volume was carried out using a weak cation exchange Sep-Pak (Accell Plus CM Light, Waters.) and eluted with saline (0.9%, 1 mL).

#### Radiolabelling of 1–7

For radiolabelling of **1–5**, benzaldehyde (48.5 μmol), the respective carboxylic acid (*p*-toluic acid (**2, 4**) or acetic acid (**1, 3, 5**), 48.5 μmol), the respective isocyanide (tert-butyl isocyanide (**4, 5**), benzyl isocyanide (**3**) or 1,1,3,3-tetramethylbutyl isocyanide (**1, 2**), 48.5 μmol), ammonium hydroxide solution (28–30%, 10 μL, 148 μmol) and [^13^N]NH_3_ (50 μL) were combined in 2,2,2-trifluoroethanol (200 μL) in a microwave tube. For radiolabelling of **6–7**, levulinic acid (48.5 μmol), the respective isocyanide (benzyl isocyanide (**6**) or tert-butyl isocyanide (**7**), 48.5 μmol), ammonium hydroxide solution (28–30%, 10 μL, 148 μmol) and [^13^N]NH_3_ (50 μL) were combined in 2,2,2-trifluoroethanol (200 μL) in a microwave tube. The mixture was heated at 120 °C for 10 min in a microwave synthesis unit with stirring. The reaction mixture was cooled to room temperature and analysed via radio-HPLC. The desired ^13^N–labelled Ugi product was isolated using semi-preparative HPLC.

## Results

### Reference standard synthesis

Based on methods developed by Thompson et al. (Thompson & Chen, 2009)*,* reference standards 1–7 were successfully synthesised and isolated. LC/MS analysis of the crude product mixtures confirmed the presence of the desired 4-CC products. Full characterisation of the final isolated products can be found in the Additional file 1.

### Radiochemistry

**Fig. 1:**
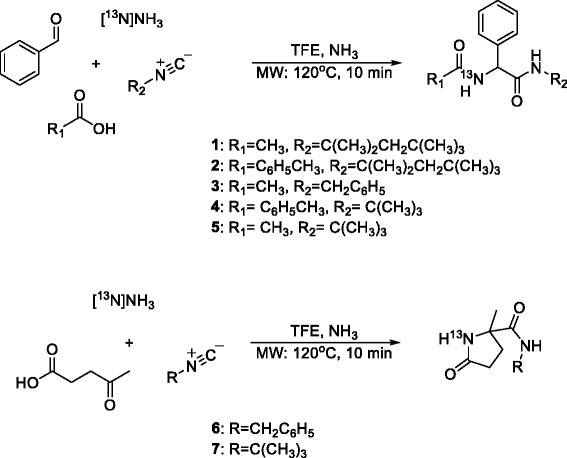
Synthesis of ^13^N–labelled α-aminoacyl amide derivatives (1–5) and γ-lactams (6–7)

Radiosynthesis of **1–5** was achieved via the Ugi reaction by combining benzaldehyde, the respective carboxylic acid and isocyanide, and carrier-added aqueous [^13^N]NH_3_ (Fig. 1). Radiosynthesis of cyclic γ-lactams **6–7** was achieved using an intra-molecular Ugi reaction by combining the ketone and carboxylic acid in a single molecule – levulinic acid - with the respective isocyanide, and carrier-added [^13^N]NH_3_ (Fig. 1). All reaction mixtures were heated using a microwave synthesis unit. Synthesis of **1** was selected as the model reaction for optimisation (Table 1). Initially, experiments were performed in a range of solvents and heated at 100 °C for 15 min. The optimum solvent was found to be TFE, affording radiochemical yields (RCY, based on radio-HPLC analysis of the crude product) of 13%, performing better than MeCN (9%), while DMF afforded no yield (Table 1, entries 1–3). Reaction time and temperature were varied, with little effect on RCY (Table 1, entries 4–8). A reaction time of 10 min at a temperature of 120 °C was found to give optimal RCY (Table 1, entry 7). However, halving the reaction time only marginally reduced the RCY from 14% to 12%. Thus, taking into account radioactive decay, a shorter reaction time of 5 min would ultimately give higher activity yield (Table 1, entry 8), and would be more practically useful in a clinical scenario. The RCY was found to have the greatest dependency on the amount of carrier ammonia added. Increasing the amount of carrier ammonia from 5 μL (74 μmol) to 10 μL (148 μmol) led to an increase in RCY from 14% to 23%, respectively (Table 1, entries 7 and 9). A further increase to 20 μL (296 μmol) resulted in a decrease in RCY to 16% (Table 1, entry 10). Under no-carrier-added conditions, the desired labelled product was not observed. Increasing the volume of the aqueous [^13^N]NH_3_ in the reaction from 50 μL to 100 μL resulted in a significant decrease in RCY.

**Table 1:**
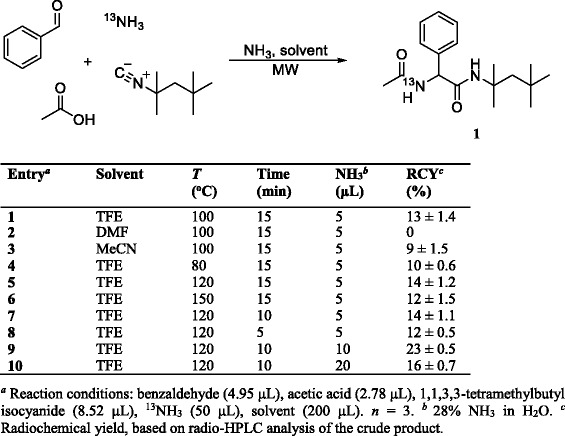
Reaction optimisation

**Fig. 2:**
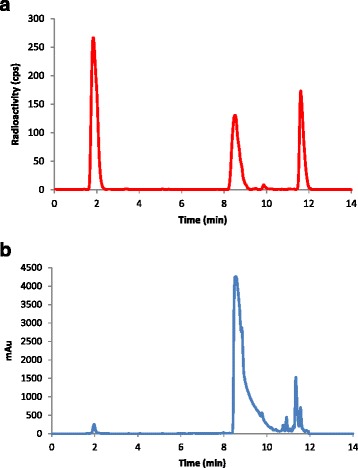
HPLC chromatogram of the crude radiolabelled product (Table 1, entry 9). **a** Radioactivity (counts per second); target compound 1 at R_t_ 11.58 min; unreacted [^13^N]NH_3_ at R_t_ 1.82 min; unknown by-product at R_t_ 8.55 min. **b** UV absorption (254 nm) of reaction 1

**Fig. 3:**
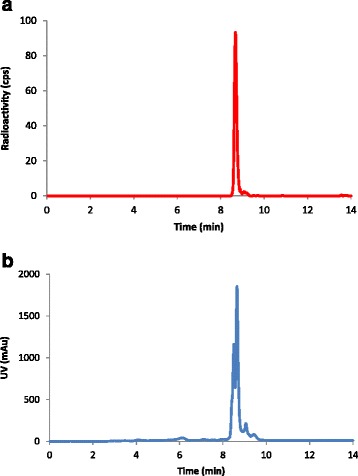
HPLC chromatogram of the isolated radiolabelled product 1. **a** Radioactivity (counts per second); **b** UV absorption (254 nm)

The established optimum conditions of **1** were applied to the radiosynthesis of a small library of compounds. ^13^N–labelling of target structures **1–7** was confirmed by the co-elution of the non-radioactive standards and radio-LC/MS analysis. The radiochemical yield of these compounds ranged from 11 to 23% (Table 2). The molar activity of [^13^N]NH_3_ used in these experiments was 2.64 ± 0.12 GBq/μmol. In addition to the target Ugi compound and un-reacted [^13^N]NH_3_, another unknown radiolabelled species was observed (Fig. 2). However, semi-preparative HPLC could be used to isolate the desired ^13^N–labelled Ugi product. To demonstrate this, isolation of **1** was carried out, achieving 96% radiochemical purity (Fig. 3) and an activity yield between 4 and 6% (based on 24 min preparation time).

**Table 2:**
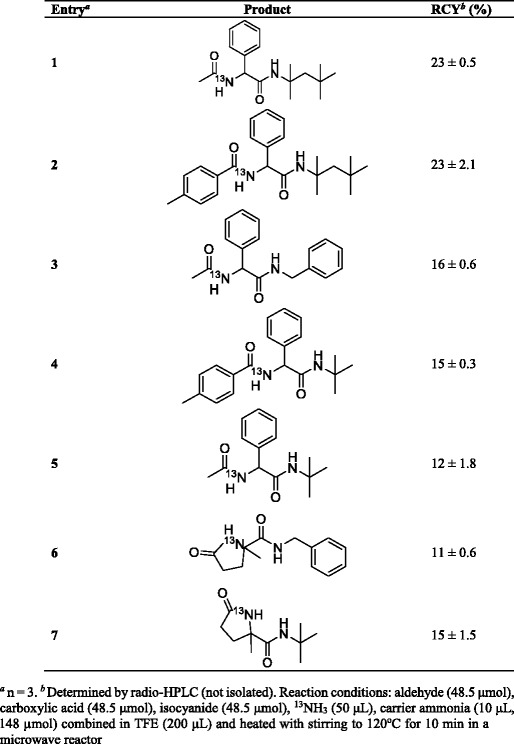
Radiolabelling of a small library of ^13^N–labelled α-aminoacyl amide derivatives and γ-lactams

**Fig. 4:**
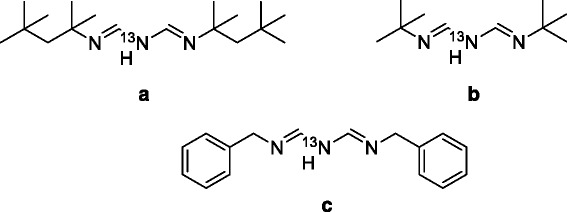
Structures of radiolabelled by-products formed from reaction of [^13^N]NH_3_ with two molecules of **a** 1,1,3,3-tetramethylbutyl isocyanide; **b** tertbutyl isocyanide and **c** benzyl isocyanide

To identify the radioactive by-products, analogous reactions using stable isotope labelling with ^14^N and ^15^N were carried out, followed by LC/MS analysis to establish a molecular weight associated with the by-products. In each case, the molecular weight corresponded to the combination of one [^14/15^N]NH_3_ molecule and two isocyanide molecules, suggesting the formation of labelled di-imine structures (Fig. 4).

## Discussion

Ammonia is rarely used as the amine component in the Ugi reaction, as it reportedly leads to low yields and extensive by-product formation. A common by-product is the six-component coupling (6-CC) product formed through participation of the solvent (usually methanol) (Ackermann et al., 2012; Kazmaier & Hebach, 2003). Thompson et al. synthesised a library of structures via the Ugi reaction using ammonia, and replacing methanol with the less nucleophilic solvent TFE suppressed formation of the 6-CC product in favour of the desired 4-CC product (Thompson & Chen, 2009). Therefore, in the present study TFE was used for all reactions. Using this strategy, reference standards **1–7** were successfully synthesised. LC/MS analysis of the crude product mixtures confirmed the presence of the desired 4-CC product, with no evidence for a 6-CC product.

The radiolabelling of structures **1–7** via the Ugi reaction using aqueous carrier-added [^13^N]NH_3_ as a precursor was successful, with RCYs ranging from 11 to 23%. Under the tested conditions, the reaction did not proceed in the absence of carrier-added ammonia and the RCY varied significantly depending on the amount of carrier ammonia added: reducing the amount of ammonia from 10 μL (2.9 equivalents) to near stoichiometric amounts (5 μL, 1.4 equivalents) reduced the RCY; increasing to 20 μL (6 equivalents), also reduced the RCY. Therefore, 10 μL was selected for all subsequent reactions. We note that under these specific high temperature, closed vessel microwave conditions, the amount of ammonia in the solution versus the gas phase (and thus unavailable for reaction) has not been quantified. The necessary addition of carrier ammonia to this reaction mixture will inherently result in a tracer with low molar activity, potentially preventing the targeting of low-abundance receptors. The detrimental effect on the RCY upon increasing the volume of aqueous [^13^N]NH_3_ suggests this reaction could be affected by the presence of water and may benefit from anhydrous conditions.

The use of microwave technology is often beneficial when rapid chemistry is required, particularly when using such short-lived isotopes as nitrogen-13. A limited number of experiments using conventional heating methods showed the target product could be obtained, but in lower RCY than that obtained using microwave heating. This indicates that the use of microwave technology is indeed effective in this case (see Additional file 1).

Despite the presence of un-reacted [^13^N]NH_3_ and radioactive by-products in the crude reaction mixture, the desired ^13^N–labelled Ugi product could be easily isolated using semi-preparative HPLC, as demonstrated for reaction **1**. The UV chromatogram of the final isolated product showed two major products co-eluting with the radioactive product. These are likely to be the non-radioactive α-aminoacyl amide Ugi product, and a structurally similar α-acyloxy amide, the product of the three-component coupling Passerini reaction between the isocyanide, carboxylic acid and aldehyde (see Additional file 1). This is consistent with our observations of the presence of the 3-component coupling Passerini product in the LC/MS analysis of the crude reaction mixture during synthesis of the non-radioactive reference standards, and in the analogous ^15^N reactions. The radioactive by-products observed in the radio-chromatograms of all reactions **1–7** were identified as ^13^N–labelled di-imines in which one [^13^N]NH_3_ molecule reacts with the electrophilic carbon of two isocyanide molecules to form the ^13^N–labelled di-imine (Fig. 4). Identification of these by-products further contributes to expanding the breadth of known radiochemistry available for radiosynthesis with [^13^N]NH_3._ Derivatisation of R_1_ and R_2_ substituents via the isocyanide and carboxylic acid moieties bearing targeting groups such as peptides, sugars or other functional groups, could enable rapid access to libraries of functionalised ^13^N–labelled compounds. Furthermore, it may be possible to label molecules of specific biological interest using this method, such as 1,4-benzodiazepine-2,5-diones which have shown promise as anticonvulsant agents, opiate receptor antagonists and as inhibitors of histone deacetylases which are linked to the pathogenesis of several cancers (Hulme et al., 1998; Loudni et al., 2007).

## Conclusion

The novel methodology presented here demonstrates an efficient strategy for quickly obtaining libraries of diverse ^13^N–labelled peptidomimetics. The Ugi reaction has been successfully adapted to radiolabel a library of α-aminoacyl amide derivatives and γ-lactams with ^13^N from cyclotron-produced aqueous [^13^N]NH_3_ precursor in a one-pot method. This is also the first report of radiosynthesis with ^13^N using microwave heating technology. This work demonstrates that despite its short half-life, complex molecules can be rapidly labelled with ^13^N and as such, ^13^N should be regarded as a viable option for labelling peptides in the future.

## Additional file

Additional file 1: Electronic Supplementary Information. (DOCX 77 kb)

## Abbreviations

4-CC: Four-component coupling
6-CC: Six-component coupling
DMF: Dimethyl formamide
PET: Positron emission tomography
RCY: Radiochemical yield
TFA: Trifluoroacetic acid
TFE: 2,2,2-trifluoroethanol
