# Supplementary material for: Convergent synthesis of 13N-labelled Peptidic structures using aqueous [13N]NH3
Source: EJNMMI Radiopharm Chem. 2017 Dec 19;2:16. doi: 10.1186/s41181-017-0035-7 (PMC5824707; doi:10.1186/s41181-017-0035-7)
Supplement: Supplementary file 1 — Electronic Supplementary Information. (DOCX 77 kb) [file 41181_2017_35_MOESM1_ESM.docx]

**Electronic Supplementary Information**

**EJNMMI Radiopharmacy and Chemistry**

**Convergent Synthesis of ^13^N-labelled Peptidic Structures Using Aqueous [^13^N]NH_3_**

**Julia E. Blower, Samuel F. Cousin and Antony D. Gee**

*School of Biomedical Engineering and Imaging Sciences, King’s College London, UK. SE1 7EH; Fax: 020 718 85442; Tel: 020 718 88366; Email:* [*antony.gee@kcl.ac.uk*](mailto:antony.gee@kcl.ac.uk)

Contents

[General Materials and Methods 2](#_Toc499146040)

[General Procedure for Preparation of Reference Compounds 2](#_Toc499146041)

[General Procedure for Preparation of Radiolabelled Compounds 3](#_Toc499146042)

[Characterisation Data for Reference Compounds 4](#_Toc499146043)

[Ugi versus Passerini structures 6](#_Toc499146044)

[References 6](#_Toc499146045)

# General Materials and Methods

Benzaldehyde (≥99%), levulinic acid (98%), *p*-toluic acid (98%), tert-butyl isocyanide (98%) and trifluoroacetic acid (99%) were purchased from Sigma-Aldrich. Benzyl isocyanide (98%) and 2,2,2-trifluoroethanol (99.8%) were purchased from Acros Organics. 1,1,3,3-tetramethylbutyl isocyanide and ammonia solution (28-30%) were purchased from Merck KGaA. Acetic acid (~100%) was purchased from AnalaR. Methanol (HPLC grade) was purchased from Fisher.

Reactions were carried out using a CEM Discover microwave synthesis unit.

^1^H-NMR and ^13^C-NMR spectra were obtained using a Bruker DRX 400 MHz spectrometer. Mass spectroscopy was performed on an Agilent Technologies 6520 Accurate-Mass Q-TOF LC/MS connected to an Agilent Technologies1200 HPLC system with UV detector and autosampler.

Radio-HPLC analysis was performed on an Agilent Technologies 1200 Series with UV detector (254 nm) and Lablogic β+ radio-detector using an Agilent Eclipse XDB-C18 column (5 μm, 4.6 × 150 mm). The following mobile phase conditions were used: solvent A: H_2_O + 0.1% TFA; solvent B: MeOH + 0.1% TFA; time:%B 0:5, 1:5, 10:95, 18:5, 23:5. Flow rate was 1 mL/min

# General Procedure for Preparation of Reference Compounds

Method was based on procedure reported by Thompson *et al* [1].

*Entries* ***1-5***

Benzaldehyde (2 mmol), carboxylic acid (2 mmol), isocyanide (2 mmol) and ammonia (excess, ~ 3 mmol) were combined in 2,2,2-trifluoroethanol (2 mL) in a microwave tube. The mixture was heated at 100 ^o^C for 30 min in a microwave synthesis unit with stirring. The reaction mixture was cooled to room temperature and the solvent was removed under vacuum using a rotary evaporator. The crude product was then filtered and washed with ice cold methanol. The desired product was then isolated using semi-preparative HPLC and lyophilised.

*Entries* ***6-7***

Levulinic acid (2 mmol), isocyanide (2 mmol) and ammonia (excess, ~ 3 mmol) were combined in 2,2,2-trifluoroethanol (2 mL) in a microwave tube. The mixture was heated at 100 ^o^C for 30 min in a microwave synthesis unit with stirring. The reaction mixture was cooled to room temperature and the solvent was removed under vacuum using a rotary evaporator. The crude product was then filtered and washed with ice cold methanol. The desired product was then isolated using semi-preparative HPLC and lyophilised.

Semi-preparative HPLC was performed on an Agilent Technologies 1200 Series with UV detector (254 nm) using an Agilent XDB-C18 column (5 μm, 9.4 × 250 mm). The following mobile phase conditions were used: solvent A: H_2_O + 0.1% TFA; solvent B: MeOH + 0.1% TFA; time:%B 0:0, 1:0, 55:100, 75:100. Flow rate was 3 mL/ min.

# General Procedure for Preparation of Radiolabelled Compounds

[^13^N]NH_3_ production

Aqueous [^13^N]NH_3_ was produced on a CTI RDS 112 biomedical cyclotron via the ^16^O(p,α)^13^N nuclear reaction. The target contained 8 mL H_2_O with 5 mM ethanol and was irradiated with 11.2 MeV protons at a beam current of 30 μA for 20 min. The irradiated solution was pumped from the cyclotron through narrow bore PEEK tubing to the radiochemistry laboratory where it is passed through an IC-OH cartridge (Maxi-Clean™, Grace Davison Discovery Sciences) conditioned with water (5 mL), to remove impurities.

Concentration of [^13^N]NH_3_ into a smaller volume (1 mL) was carried out by loading the solution on to a weak cation exchange Sep-Pak (Accell Plus CM Light, Waters.) conditioned with water (5 mL), and eluted with saline (0.9%, 1 mL).

*Entries* ***1-5***

Benzaldehyde (48.5 μmol), carboxylic acid (48.5 µmol), isocyanide (48.5 μmol), ammonium hydroxide solution (28-30%, 10 µL, 148 µmol) and [^13^N]NH_3_ (50 μL) were combined in 2,2,2-trifluoroethanol (200 μL) in a microwave tube. The mixture was heated at 120 ^o^C for 10 min in a microwave synthesis unit with stirring. The reaction mixture was cooled to room temperature and analysed via radio-HPLC. Isolation of **1** was carried out using semi-preparative radio-HPLC.

Semi-preparative radio-HPLC was performed on an Agilent Technologies 1200 Series with UV detector (254 nm) and Lablogic β+ radio-detector using an Agilent Eclipse XDB-C18 column (5 µm, 9.4 × 250 mm). The following mobile phase conditions were used: solvent A: H_2_O + 0.1% TFA; solvent B: MeOH + 0.1% TFA; time (min):%B 0:50, 1:50, 5:95, 10:95, 15:5, 18:5. Flow rate was 3 mL/min.

*Entries* ***6-7***

Levulinic acid (48.5 µmol), isocyanide (48.5 μmol), ammonium hydroxide solution (28-30%, 10 µL, 148 µmol) and [^13^N]NH_3_ (50 μL) were combined in 2,2,2-trifluoroethanol (200 μL) in a microwave tube. The mixture was heated at 120 ^o^C for 10 min in a microwave synthesis unit with stirring. The reaction mixture was cooled to room temperature and analysed via radio-HPLC.

*Preparation of* ***1*** *using conventional heating methods*

Benzaldehyde (48.5 μmol), carboxylic acid (48.5 µmol), isocyanide (48.5 μmol), ammonium hydroxide solution (28-30%, 5 µL, 74 µmol) and [^13^N]NH_3_ (50 μL) were combined in 2,2,2-trifluoroethanol (200 μL) in a sealed reaction vessel. The mixture was heated at 100 ^o^C for 15 min. The reaction mixture was cooled to room temperature and analysed via radio-HPLC. The RCY was 8% (c.f. microwave heating: Table 1, entry 1, RCY = 13%).

# Characterisation Data for Reference Compounds

**2-acetamido-2-phenyl-*N*-(2,4,4-trimethylpentan-2-yl)acetamide (1);** white solid; ^1^H NMR (CDCl_3_, 400 MHz): δ 7.31-7.20 (m, 5H), 6.94 (bd, *J*= 6.7 Hz, 1H), 5.66 (bs, 1H), 5.30 (d, *J*= 6.8 Hz, 1H), 1.94 (s, 3H), 1.55 (q, *J*=14.9 Hz, 2H), 1.27 (s, 6H), 0.76 (s, 9H);^13^C NMR (CDCl_3_, 400 MHz): δ 169.6, 168.6, 138.4, 128.9, 128.7, 128.2, 55.9, 52.1, 31.2, 31.4, 29.02, 28.4, 23.2.

LC/MS (ESI+): calculated for C_18_H_29_N_2_O_2_ [M+H]^+^: 305.2224; found 305.2225.

**4-methyl-*N*-*(*2-oxo-1-phenyl-2-((2,4,4-trimethylpentan-2-yl)amino)ethyl)benzamide (2);** white solid; ^1^H NMR (CDCl_3_, 400 MHz): δ 7.71 (d, *J*= 8.2 Hz, 2H), 7.68 (bs, 1H), 7.43-7.18 (m, 7H), 5.72 (bs, 1H), 5.50 (d, *J*= 6.3 Hz ,1H), 2.35 (s, 3H), 1.60 (q, *J*= 15 Hz, 2H), 1.32 (s, 6H), 0.79 (s, 9H);^13^C NMR (CDCl_3_, 400 MHz): δ 168.66, 166.44, 142.12, 138.66, 131.10, 129.16, 129.00, 128.22, 127.36, 127.19, 57.91, 55.86, 52.16, 31.45, 31.26, 29.08, 28.50, 21.48

LC/MS (ESI+): calculated for C_24_H_33_N_2_O_2_ [M+H]^+^: 381.2537; found 381.2549.

**2-acetamido-*N*-benzyl-2-phenylacetamide (3);** white solid; ^1^H NMR (CDCl_3_, 400 MHz): δ 7.42-7.13 (m, 10H), 7.03 (bd, *J*= 6.7 Hz, 1H), 6.57 (bs, 1H), 5.62 (d, *J*=7.1 Hz,1H), 4.41 (qd, *J*= 16.0, 5.5 Hz, 2H), 1.97 (s, 3H);^13^C NMR (CDCl_3_, 400 MHz): δ 170.03, 169.73, 138.07, 137.48, 129.03, 128.68, 128.41, 127.55, 127.53, 127.29, 57.02, 43.76, 23.13.

LC/MS (ESI+): calculated for C_17_H_19_O_2_N_2_ [M+H]^+^: 283.1441; found 283.1461.

***N*-(2-(tert-butylamino)-2-oxo-1-phenylethyl)-4-methylbenzamide (4);** white solid; ^1^H NMR (CDCl_3_, 400 MHz): δ 7.75 (d, *J*=7.9 Hz, 2H), 7.66 (bs, 1H), 7.48 (d, *J*=7.4 Hz, 2H), 7.38-7.22 (m, 5H), 5.84 (bs, 1H), 5.63 (d, *J*=6.5 Hz, 1H), 2.41 (s, 3H), 1.32 (s, 9H);^13^C NMR (CDCl_3_, 400 MHz): δ 169.22, 166.50, 142.17, 138.80, 131.04, 129.17, 128.99, 128.19, 127.26, 127.20, 57.47, 51.90, 28.57, 21.48.

LC/MS (ESI+): calculated for C_20_H_25_N_2_O_2_ [M+H]^+^: 325.1911; found 325.1941.

**2-acetamido-*N*-(tert-butyl)-2-phenylacetamide (5);** white solid; ^1^H NMR (CDCl_3_, 400 MHz): δ 7.40-7.31 (m, 5H), 6.88 (bd, *J*=6.2 Hz, 1H), 5.51 (bs, 1H), 5.38 (d, *J*=7.1 Hz, 1H), 2.03 (s, 3H), 1.31 (s, 9H);^13^C NMR (CDCl_3_, 400 MHz): δ 169.48, 169.02, 138.57, 129.03, 128.29, 127.27, 57.16, 51.91, 28.54, 23.27.

LC/MS (ESI+): calculated for C_14_H_21_N_2_O_2_ [M+H]^+^: 249.1598; found 249.1618.

***N*-benzyl-2-methyl-5-oxopyrrolidine-2-carboxamide (6);** colourless oil; ^1^H NMR (CDCl_3_, 400 MHz): δ 7.38-7.28 (m, 5H), 6.75 (bs, 1H), 6.68 (bs, 1H), 4.46 (s, 2H), 2.55-2.49 (m, 1H), 2.42 (t, *J*=7.8 Hz, 2H), 2.15-2.07 (m, 1H), 1.58 (s, 3H);^13^C NMR (CDCl_3_, 400 MHz): δ 178.91, 173.80, 137.65, 128.86, 127.82, 127.81, 63.54, 43.90, 33.66, 29.97, 25.42.

LC/MS (ESI+): calculated for C_13_H_17_O_2_N_2_ [M+H]^+^: 233.1285; found 233.1286.

***N*-(tert-butyl)-2-methyl-5-oxopyrrolidine-2-carboxamide (7);** colourless oil; ^1^H NMR (CDCl_3_, 400 MHz): δ 7.21 (bs, 1H), 6.12 (bs, 1H), 2.78 (m, 1H), 2.64 (m, 1H), 2.48 (m, 1H), 2.08 (m, 1H), 1.53 (s, 3H), 1.37 (s, 9H);^13^C NMR (CDCl_3_, 400 MHz): δ 176.95, 173.17, 64.16, 51.69, 37.81, 30.10, 28.50, 25.29.

LC/MS (ESI+): calculated for C_10_H_19_O_2_N_2_ [M+H]^+^: 199.1441; found 199.1456.

# Ugi versus Passerini structures

General structure of a Ugi reaction product (A) versus a Passerini reaction product (B).

# References

1. M. J. Thompson and B. Chen, *J. Org. Chem.,* 2009, **74**, 7084-7093.
